# Why should I when no one else does? A review of social norm appeals to promote sustainable minority behavior

**DOI:** 10.3389/fpsyg.2024.1415529

**Published:** 2024-09-11

**Authors:** Anna Schorn

**Affiliations:** Department of Communication and Media Research, University of Zurich, Zürich, Switzerland

**Keywords:** minority behavior, nudging, social norm appeals, sustainability, social norms, social change, backfire effects, environmental psychology

## Abstract

Social norm appeals have been proven successful in promoting sustainable behavior that most people engage in. However, research on the effectiveness of social norm appeals in promoting sustainable behavior performed by a numerical minority of people is lacking. This systematic review aimed to examine empirical studies that applied social norm appeals and to elaborate on how social norm appeals could be effectively designed to foster sustainable minority behaviors. Thirty-six articles, including 54 studies, applying social norm interventions to promote sustainable minority behavior were compiled and discussed, with a particular focus on the methodology and operationalization of social norm appeals. The results showed that static descriptive minority social norm appeals might not be effective in promoting sustainable behavior. Nevertheless, there appeared to be differences depending on the strength of the norm and the environmental attitudes of the population. However, using injunctive and dynamic descriptive social norm appeals appear promising approaches because these appeals are less prone to undesirable effects. Nevertheless, it could be problematic if injunctive and descriptive social norm appeals are not aligned, but results are inconclusive. For practitioners, emphasizing social change and highlighting majority approval are simple, low-cost strategies with great potential to induce compliance and encourage sustainable minority behavior without running the risk of backfire effects.

## Introduction

Mitigating the climate crisis is a global challenge facing all individuals, nations, and economic sectors (United Nations, 2015). Despite international arrangements, such as the Paris Agreement to combat climate change and its negative impacts, anthropocentric contributions to greenhouse gas emissions are far beyond the defined targets (Fell and Traber, 2020; IPCC, 2021). Households are estimated to be responsible for up to 72% of global emissions (Hertwich and Peters, 2009). Therefore, changing individual consumption behavior remains a critical, contemporary ambition (Fell and Traber, 2020).

In behavioral sciences, one of the most important interventions to change behavior in general and to motivate sustainable behavior in particular is the use of social norm appeals (Rhodes et al., 2020; Cialdini and Jacobson, 2021). Social norm interventions can be subtle, simple, low-cost, and effective ways to encourage compliance (Mortensen et al., 2019; Rhodes et al., 2020). Social norm appeals attempt to change behavior by modifying the prevailing view that a particular behavior is more prevalent or has gained wide approval in a certain social context (Mortensen et al., 2019; Rhodes et al., 2020; Cialdini and Jacobson, 2021).

According to the focus theory of normative conduct, descriptive social norm appeals (DSNAs) provide information about the proportion of people who engage in the target behavior, while injunctive social norm appeals (ISNAs) describe the proportion of people approving of the behavior within a reference group (Cialdini et al., 1991; Goldstein et al., 2008; Schultz et al., 2007). Several meta-analyses have shown that both types of social norm appeals are effective in promoting sustainable behavior when implemented and approved by a numerical majority of people (Cialdini et al., 1991; Abrahamse and Steg, 2013; Poškus, 2016; Farrow et al., 2017; Rhodes et al., 2020).

However, measures to protect the environment and sustainable habits are often new behaviors that may only be exhibited initially by a numerical minority of people (e.g., Brechin and Bhandari, 2011; European Commission, 2020, 2021; Passafaro, 2020; de Groot, 2022). When the targeted behavior is not prevalent, DSNAs run the risk of undesirable boomerang or backfire effects when people learn that their (undesirable) behavior is the norm (Reno et al., 1993; Loschelder et al., 2019). In this case, normative information can produce the opposite of what a communicator intends (Cialdini, 2003; Schultz et al., 2007; Richter et al., 2018; Berger, 2021). However, recent studies have used dynamic DSNAs to present behavior as a growing trend that more and more people are following to prevent such undesirable effects (Sparkman and Walton, 2017; Mortensen et al., 2019).

At the same time, environmental issues have received increasing attention in politics and the mass media, and different studies have shown a high awareness of climate change in large parts of the world population (Brechin and Bhandari, 2011; Lee et al., 2015; Baiardi and Morana, 2021; Andre et al., 2024). Most people see climate change and sustainability as important problems, and the majority appear to have realized that something must be done to protect the environment (Baiardi and Morana, 2021; Economist Intelligence Unit, 2021; Andre et al., 2024). Therefore, people seem to approve of sustainable behavior in general and of specific actions, but they do not yet adapt their own behavior to the same extent. Thus, the initial situation for new sustainable behaviors would often include a collective injunctive majority social norm (most people approve of sustainable behaviors) and a collective descriptive minority social norm (only a few people engage in the behavior).

Given these circumstances, the purpose of the present research was to determine how social norm appeals can be used effectively to activate social norms with the aim of promoting sustainable behaviors performed by a numerical minority of people. To do so, this systematic review investigated previous empirical studies that applied social norm appeals to promote sustainable minority behaviors and elaborates on how they could be effectively designed to foster sustainable minority behaviors. Several reviews and meta-analyses have already focused on social norm interventions in general (Rimal and Lapinski, 2015; Chung and Rimal, 2016; Legros and Cislaghi, 2020; Lutkenhaus et al., 2023) and to promote environment-friendly behaviors (Bergquist et al., 2020; Miller and Prentice, 2016; Poškus, 2016; Farrow et al., 2017; Yamin et al., 2019; Rhodes et al., 2020; Saracevic and Schlegelmilch, 2021; Helferich et al., 2023). However, they did not explicitly focus on minority behaviors which is why the results have only limited applicability within the context of sustainability specifically. Moreover, this literature review provides a qualitative focus and discussion of methodological variances which complements meta-analytical studies. Since there is great variability in the study designs, a narrative review can be particularly useful because the existing studies are not homogeneous in terms of design, measures, participants, interventions, control groups and outcomes. If these differences are taken into account, only little studies remain that can be meaningfully compared with each other due to a similar design. In addition, in the context of minority behavior, the state of research suggests that such interventions may be ineffective, which is why it is enriching to discuss under which exact circumstances social norm appeals can be used effectively in the context of minority behavior or not. Complementary to other narrative reviews that provide informal information for practitioners and policymakers (Sparkman et al., 2020a) or a concise, outcome-oriented research overview (Cialdini and Jacobson, 2021), this review compares and discusses the methods and operationalizations with regard to the respective results.

## Theoretical background

The idea that the behavior of individuals is influenced by the behavior of their social group has a long tradition in research (Fishbein and Ajzen, 1975; Schwartz, 1973; Sherif, 1936). However, social influence research has entered a new era with the research on social norms by Cialdini et al., whose focus theory of normative conduct is based on the premise that social norms powerfully and systematically influence human behavior (Cialdini et al., 1990, 1991). According to the theory, there are descriptive norms that reflect the typical or normal behavior of people and injunctive norms that reflect what behavior is commonly desirable or approved (Cialdini et al., 1991). Descriptive norms can influence behavior based on social proof because they indicate behavior that has proven to be effective for others (Jacobson et al., 2020). Injunctive norms can influence behavior by creating social pressure to conform because they show what behavior a social group approves or expects. According to focus theory, individuals conform to the focal or salient norm even when other types of norms dictate a behavior contrary to the target behavior (Cialdini et al., 1991). This means that social norms can be activated or made salient through social norm appeals so that they can serve as guides for behavioral decisions.

### Descriptive social norm appeals

Descriptive social norms refer to what other people do or the behaviors they engage in. Typically, they characterize the perception of what most people do within a reference group (Cialdini et al., 1991). Descriptive norms can be used by individuals as evidence of how (most) people behave and, therefore, of what will likely be effective behavior in a certain context. In this way, descriptive norms work heuristically as shortcuts when people imitate what most people do because that is likely to be effective for a given situation (Cialdini et al., 1990).

To modify the perceived descriptive norms, DSNAs indicate how high the frequency of occurrence of a target behavior is within a certain reference group (e.g., “Nearly 25% of guests choose to reuse their towels each day”). However, when individuals learn that only a small number of people engage in the target behavior, this cannot serve as social proof and should not encourage compliance. In this case, DSNAs can lead to backfire effects that suppress the target behavior.

Within DSNAs, a further distinction can be made between static and dynamic DSNAs. Static DSNAs report the proportion of people currently performing the behavior, while dynamic DSNAs highlight trends and social change (Sparkman and Walton, 2017; Mortensen et al., 2019). Dynamic DSNAs are specifically studied in the context of minority behavior because they can prevent the undesirable effects of static DSNAs that often occur in the context of sustainable behavior.

### Injunctive social norm appeals

Injunctive social norms constitute the moral rules of a group and motivate actions by promising social rewards or creating a fear of social sanctions for them (Cialdini et al., 1991). However, people systematically underestimate the approval of different environmental behaviors in the population and have a misperception of injunctive norms (Nolan, 2021; Wolf et al., 2023; Andre et al., 2024). ISNAs can adjust these misperceptions and increase perceived injunctive norms (e.g., “85% of the student sample approves of other students who engage in energy conservation”). However, when looking at ISNAs, it is evident that they are not consistently defined and applied (Shulman et al., 2017; Schorn et al., 2023).

In experimental studies, some authors used ISNAs that stated directly what behavior should be performed with regard to a reference group (prescriptive ISNAs; Melnyk et al., 2013; White and Simpson, 2013; He et al., 2019), while others only referred to whether the behavior had the approval of the reference group (approving ISNAs; Smith and Louis, 2008; de Groot and Schuitema, 2012; Smith et al., 2012; Bonan et al., 2020; Ge et al., 2020; Schorn and Wirth, 2023). In some studies, an ISNA was simply an appeal directing people on what to do (e.g., “Choose a sustainable cup!”) (Mollen et al., 2013; Bergquist and Nilsson, 2019; Loschelder et al., 2019; Poškus et al., 2019). However, these appeals made without a reference group should not be considered ISNAs because they might not have activated the perceived injunctive norms, as the norm was not directly stated (cf. Cialdini and Jacobson, 2021). Therefore, they may not build up social pressure or the fear of social sanctions but rather activate the moral obligation to act environmentally friendly.

Overall, majority ISNAs seem to be suitable for promoting environment-friendly behavior (Rhodes et al., 2020). Nevertheless, due to the described methodological differences, it is important to look closely at the operationalization of ISNAs if their effectiveness is to be assessed, particularly in the context of minority behavior. Moreover, the few studies investigating ISNAs in the context of minority behavior typically include not only ISNAs but also DSNAs (Schultz et al., 2008; de Groot and Schuitema, 2012; Smith et al., 2012). Therefore, this interaction of ISNAs and DSNAs must be considered when researching sustainable behaviors.

### Conflicting social norm appeals

Individuals often have prevalent positive attitudes on environmental topics and seem to approve of sustainable behavior and specific actions, but they do not adapt their own behavior to the same extent (European Commission, 2020; Baiardi and Morana, 2021; Economist Intelligence Unit, 2021). For example, around 90% of Europeans stated that people should be educated on how to behave more sustainably and that authorities and industry should make greater efforts to reduce plastic waste, but only one-third of the respondents avoided buying over-packaged products (European Commission, 2020). Such attitude–behavior gaps indicate conflicting social norms on a higher level because most people seem to approve of sustainable behaviors, but due to different barriers, only a small number of people adopt the corresponding behaviors (cf. Gifford, 2011; Lacroix et al., 2019). Thus, the initial situation for sustainable behaviors can involve a collective injunctive majority but a descriptive minority social norm.

In the context of sustainable behavior, it is therefore important to not only compare DSNAs and ISNAs but also investigate how different social norm appeals influence each other. People could experience an inner conflict or cognitive dissonance that could suppress the desired behavior when they experience that the usual behavior does not correspond to what should be done (cf. Thøgersen, 2008; Bonan et al., 2020; Jacobson et al., 2020). Accordingly, research on social norm conflict has argued that it can be counterproductive if ISNAs do not match DSNAs, and vice versa (Smith et al., 2012; Ge et al., 2020). However, in this context, there are again differences in the way the studies were conducted and it is important to consider exactly which operationalization the researchers used to arrive at which result.

## Materials and methods

This study aimed to determine how social norm appeals can be used effectively for promoting sustainable minority behavior. To do so, empirical studies investigating effects of social norm appeals were reviewed. Survey-only studies were not included because they generally did not involve social norm appeals or interventions and only measured perceived social norms. To make the results comparable, the social norm message should state that the promoted behavior is performed by a numerical minority of people within a reference group in a static or dynamic component, either numerically or in words referring to a share. This is because social norm appeals may affect persuasive outcomes due to a change in the projected commonness of behavior (Sparkman and Walton, 2017; Loschelder et al., 2019; Mortensen et al., 2019). Other normative messages may not affect perceived social norms and thus operate differently (Poškus et al., 2019). Therefore, studies in which social norm appeals were formulated negatively were also excluded. Consequently, the following criteria were used to decide whether a study would be included in the analysis:

- The aim of the study was to promote sustainable behaviors or attitudes.
- The promoted behavior was performed by a numerical minority of people (< 50%) within a reference group (minority behavior).
- There was an intervention or manipulation of minority descriptive, injunctive, or both types of social norm appeals (experimental study).
- The message verbally stated in a social norm appeal that the promoted behavior was performed by a numerical minority of people in a static or dynamic component (social norm appeal).
- The social norm appeal was phrased positively. The focus must be on the minority performing the target behavior and not on the majority *not* performing the behavior.

**Figure 1:**
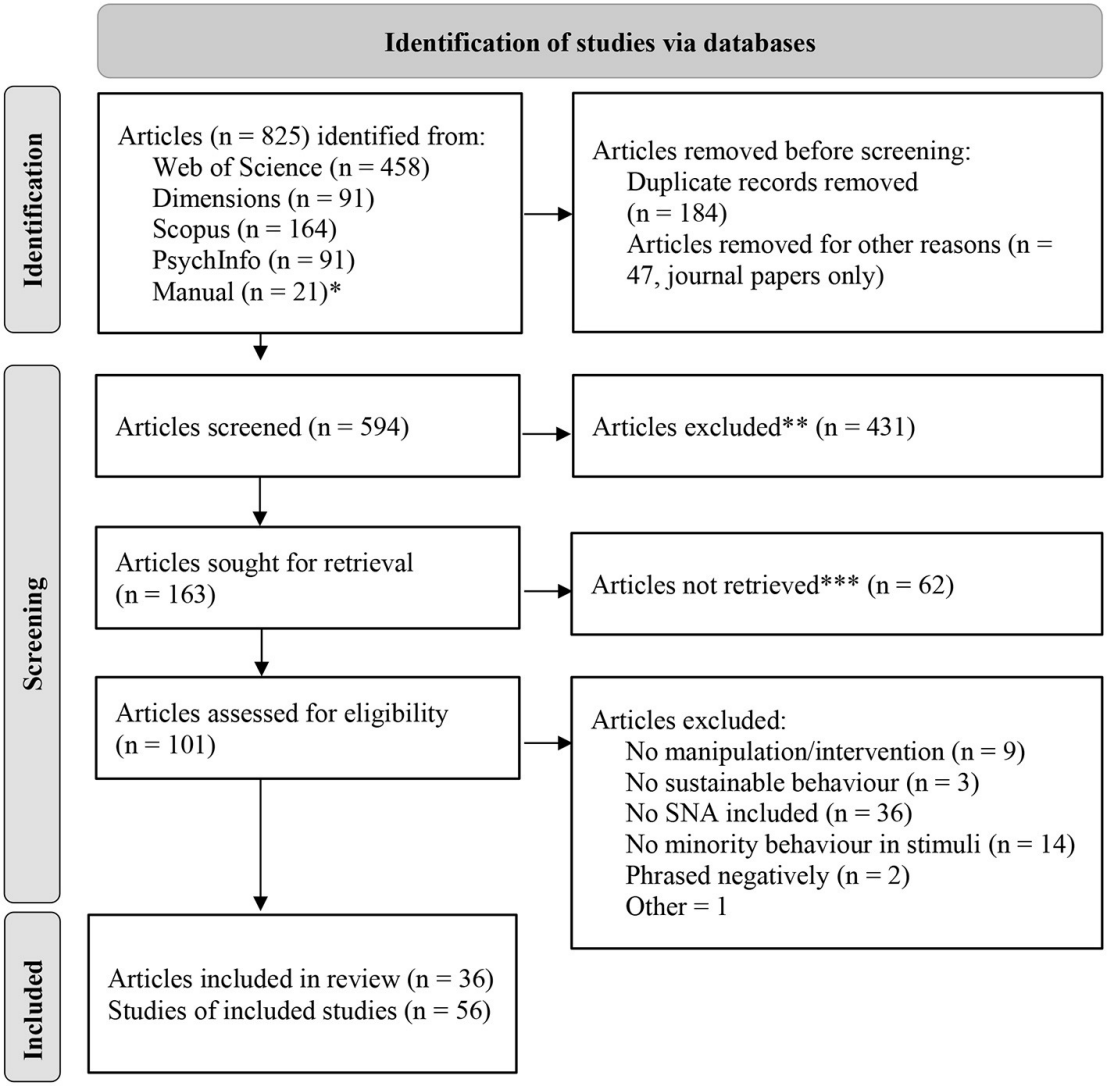
PRISMA 2020 flow diagram for new systematic reviews (Page et al., 2021). ^*^The manually added articles also include new studies that were published during the review and publication process and were added subsequently. ^**^Articles from journals not relevant to the topic have been excluded (e.g., biological journals, zoology, animal research, health journals etc.). ^***^The title was a strong indicator that (1) the study did not address sustainable behavior, (2) the study did not address minority behavior, (3) there was no intervention or manipulation of minority descriptive, injunctive, or both types of SNAs (experimental study), or (4) the message did not state that the promoted behavior or attitude was held by a minority in a dynamic or static component. (5) The SNA was phrased negatively.

Various databases were searched (e.g., Web of Science, Scopus, Dimensions, and PsychInfo) using the following search string: [(environmentally friendly) OR (green consumption) OR (environmental) OR (conservation) OR (environmentally-friendly) OR (sustainable)] AND [(injunctive OR descriptive OR trending OR dynamic) AND (norm)] AND (experiment OR intervention) NOT (survey) in the abstracts, titles, and keywords. The references of the included articles were also searched for additional studies. The process from identification to inclusion is summarized in the PRISMA diagram (Page et al., 2021; see Figure 1).

## Results

Following this procedure, 36 articles, including 54 studies, using social norm appeals were identified (Table A1). The articles were published between 2007 and 2024, with 24 published in the last five years, demonstrating that this is a novel and steadily growing field. In the following, a descriptive overview of the individual studies included in the papers will be provided before the results are reviewed and discussed with regard to the effects.

### Descriptive overview

The majority of studies were (online) experiments (>78%) but the proportion of field experiments was also relatively high at approximately 20%. Most studies were conducted in North America (~46%) and Europe (~43%), whereas only a few were conducted in other parts of the world. The studies were mainly in the areas of sustainable diet (>36%), followed by energy and water conservation (14%), sustainable consumption (14%), waste prevention (9%), voluntary carbon offsets (7%) and transportation (5%). Most studies focusing on minority behavior referred to descriptive norms and DSNAs, whereas only a few studies examined different characteristics of ISNAs (Schultz et al., 2008; de Groot and Schuitema, 2012; Smith et al., 2012; Lalot et al., 2018, 2019). Most studies focused on dynamic DSNAs and compared them vs. a control group (~29%) or a static DSNAs (~27%). More than a third of the studies contrasted minority with majority behavior (~36%) and few studies compared the strength of different minority DSNAs. Moreover, the studies differed in their dependent variables: Field experiments typically observed actual behavior (e.g., Berger, 2021; Loschelder et al., 2019; study 1; Richter et al., 2018), while lab and online experiments typically measured behavioral intentions (e.g., Smith et al., 2012; Aldoh et al., 2021; de Groot et al., 2021; Aruta, 2022) or the interest in the behavior (e.g., Sparkman and Walton, 2017). Moreover, some of the studies measured effects on perceived social norms (e.g., Lapinski et al., 2017; Reynolds-Tylus et al., 2019). In contrast to the field experiments, additional variables were often measured in the online experiments, which is beneficial if the mechanisms of action of social norm appeals are considered (e.g., Sparkman et al., 2020b). Most studies have used student or convenience samples and only a few studies have worked with quasi-representative samples, which is partly due to the reference groups used (see Table A1).

### Procedure

The review process followed three steps. First, the articles were reviewed inductively for commonalities and differences, with a particular focus on methodology and operationalization. Based on this initial assessment of the current state of research, various categories were determined in the second step to which the research questions and findings from the articles could be assigned. These categories were the basis for developing the guiding questions that were explicitly or implicitly addressed in the articles. For example, some of the questions were not directly examined in the studies, but they were raised in the discussion of the respective articles. Therefore, the formulation of such guiding questions was important to relate the studies to each other and to discuss the results accordingly.

Overall, six guiding questions were identified in this initial screening. Studies applying minority DSNAs raised the questions of whether there were differences in the effect depending on the strength of the social norm appeal (Q1) or sample characteristics (Q2) and under which circumstances they may lead to backfire effects (Q3). Studies using dynamic DSNAs examined whether such negative effects caused by static DSNAs could be prevented by highlighting a trend in behavior (Q4). Studies applying ISNAs have raised the question of whether their effect was also influenced by the strength of the social norm appeal (Q5). Studies using descriptive and ISNAs, which could be either congruent or conflicting, raised the question of how this alignment affected their impact (Q6). In the third step, the results of the studies were compiled and compared with reference to these guiding questions.

### Effects of descriptive minority social norm appeals

There are various studies on the effects of minority DSNAs (see Table A1). On the one hand, experiments examine whether there are differences on persuasive outcomes depending on the strength of (majority and minority) DSNAs (Q1). Among these, there are studies that suggest that the effects differ depending on the environmental dispositions of the participants (Q2), which can also be decisive for the occurrence of backfire effects (Q3). On the other hand, many experiments compare dynamic DSNAs against either static DSNAs or a control group without DSNAs (Q4).

#### Q1: How do the effects of static descriptive social norm appeals depend on the strength of the social norm appeal?

In their early research on DSNAs, Demarque et al. (2015) found that both descriptive majority (70%) and minority DSNAs (9%) increased the number of ecological products sold. However, their second experiment with a different population confirmed these results only for majority DSNAs (< 50%). Similar to the latter result, Aruta (2022) showed that minority DSNAs are less effective than majority DSNAs. Furthermore, Aldoh et al. (2021), Berger (2021), Loschelder et al. (2019), Mortensen et al. (2019), Richter et al. (2018), Schorn and Wirth (2023, 2024), Shealy et al. (2018), and Sparkman and Walton (2017) concluded that static minority DSNAs did not have positive effects compared with the control groups. Moreover, Lapinski et al. (2017) showed that a minority DSNA (3%) lowered the perceived prevalence of the behavior compared with a majority DSNA (90%), which in turn lowered the behavioral intention but not the attitude toward the behavior. Similarly, Reynolds-Tylus et al. (2019) revealed that a minority DSNA (27%/32%) lowered the perceived prevalence of the behavior compared with a majority DSNA (73%/68%), which also lowered behavioral intention. However, neither Lapinski et al. (2017) nor Reynolds-Tylus et al. (2019) reported direct effects on persuasive outcomes.

Overall, minority DSNAs do not seem to be effective in promoting sustainable behavior, regardless of their strength. Some of the reviewed studies even indicated that the use of minority DSNAs could backfire, meaning that the minority DSNAs performed worse than the control groups without any normative information (Schultz et al., 2007; Richter et al., 2018; Mortensen et al., 2019; Berger, 2021). However, six of the reviewed articles concluded, either explicitly or implicitly, that there may have been differences in the effects of minority DSNAs due to sample characteristics, which could be a crucial factor when analyzing the effects of social norm appeals.

#### Q2: How do the effects of static descriptive minority social norm appeals depend on sample characteristics?

Demarque et al. (2015) attributed the varying results in different studies to the fact that samples with different characteristics were used (general population of university students vs. business students). In their first lab experiment, they did not find differences between a minority DSNA and a majority DSNA. Therefore, the strength of the DSNA was further differentiated in their second experiment (1%, 9%, 70%, and 90%). In contrast to the first experiment, consumers in the majority conditions (>50%) bought and spent more money on green products than those in the minority conditions (< 50%). Due to the sample differences from the first experiment, they suggested that the DSNAs could have had different effects depending on the level of environmental awareness of the sample: Differences between majority and minority DSNAs may only have occurred among the less environmentally concerned business students, while in the more environmentally concerned group of all students the mere activation of social norms may have been sufficient (vs. the control group).

Richter et al. (2018) posted signs using seven DSNAs, ranging from 4% to 91%, to promote sustainable seafood in a field experiment conducted in Norway and Germany. They did not find significant differences when all social norm appeals were compared, but they found intercountry differences with Norwegian supermarkets selling proportionately more sustainable seafood. When they divided the DSNAs by minority (< 50%) and majority (>50%), only a sign reminding consumers of the possibility of buying sustainable seafood (control group) in Norway showed an effect. In Germany, however, they found a significant decrease in sustainable seafood sales for minority DSNAs. Moreover, they showed that the total amount of seafood sold increased significantly during the experiment, which confirmed that the intervention had an effect but not the intended one. In accordance with Demarque et al. (2015), they concluded that in the case of minority behavior, it was risky to emphasize this fact through a DSNA and that there might be differences depending on the population.

Aruta (2022) investigated gender differences between a majority (80%) and a minority DSNA (20%), as women seemed to have stronger pro-environmental attitudes than men. Men in the majority DSNA condition reported a higher intention to reduce their plastic use than men in the minority condition. In the minority condition, women reported higher levels of plastic reduction intention than men did. There was no difference between women in the minority condition and women in the majority condition, indicating that there were fewer differences between minority and majority DSNAs for people with higher pro-environmental attitudes. However, Aruta did not control for environmental awareness. Nevertheless, a recent study by Schorn and Wirth (2024) investigated DSNAs and ISNAs and environmental dispositions in two-wave studies. In two studies, they found no interaction between environmental awareness or personal norms and the social norm appeals.

de Groot et al. (2021) investigated DSNAs (20% vs. 80%) with personal norms as a moderator. Personal norms reflect feelings of moral obligation to do “the right thing” and are self-based expectations for behavior that result from an individuals' internalized values (Cialdini et al., 1991). For participants with weak personal norms, a majority DSNA resulted in stronger behavioral intentions than a minority DSNA. For medium and strong personal norms, no differences were found depending on the DSNA. Correspondingly, Carfora et al. (2022) investigated the moderating role of intrinsic motivation, which they operationalized as similar to personal norms. They concluded that a (dynamic) DSNA seemed to be particularly effective among people with relatively weak intrinsic motivation. By contrast, Kácha and van der Linden (2021) found no significant interactions between moral norms and minority vs. majority DSNAs. Nevertheless, they suggested that this could be due to the moral norms being measured prior to the stimulus, and that this activation of moral norms could have overridden the effects of the different DSNAs.

Taken together, Aruta (2022), Carfora et al. (2022), de Groot et al. (2021), Demarque et al. (2015), and Richter et al. (2018) showed that sample characteristics could influence the effects of social norm appeals. Generally, DSNAs appear to have greater effects on individuals with weaker pro-environmental attitudes or on populations that have been less likely to engage in sustainable behaviors. For individuals with strong pro-environmental attitudes, an appeal emphasizing sustainable behavior in general seems sufficient to evoke conformity. Moreover, Richter et al. (2018) concluded that minority DSNAs could even lead to backfire effects when pro-environmental attitudes were already low. Nevertheless, Schorn and Wirth (2024) did not find positive or negative effects of social norm appeals depending on their participant's environmental dispositions.

#### Q3: Under which conditions do static descriptive minority social norm appeals backfire?

Several studies have investigated minority behavior and provided inconclusive results regarding backfire effects. Player et al. (2018) used a minority DSNA (25%) and asked people to turn off their engines when the barriers were down. However, they did not find significant differences compared with the control group, and descriptively, this led to a positive effect rather than a backfire effect. Kácha and van der Linden (2021) compared majority (83%) and minority (17%) DSNAs and found no differences when feelings of obligation to do the right thing (moral norms) were measured before the stimulus. However, when moral norms were not activated by a pre-stimulus measurement, the minority appeal was less effective. Conversely, this means that static minority DSNAs may not lead to backfire effects when moral norms are simultaneously activated.

Lee and Liu (2023) found no difference in the intention to get a flu shot between a minority DSNA (35%) and a control group without social norm appeal. However, when considering meat consumption, the participants who viewed a minority DSNA (35%) had a significantly lower intention to reduce red meat consumption than those who were not exposed to any messages. Therefore, backfire effects occurred only for environmental behavior but not for health behavior, which could be due to getting a flu shot being a private and unseen behavior and meat consumption being a social behavior. When considering public contexts, the sensitivity to social proof and social pressure may be increased as compared to private behaviors which can enhance the susceptibility to social norm appeals (cf. Habib et al., 2021). At the same time, this would also lead to an increased susceptibility for backfire effects of minority DSNAs.

In a field experiment, Berger (2021) investigated how minority DSNAs could be used to promote reusable mugs instead of disposable paper cups. A constant appeal (“use mugs instead of paper cups”) was supplemented with descriptive numbers: Individuals were informed that either 22% (weak minority DSNA) or 41% of customers used reusable cups (strong minority DSNA). These numbers were updated weekly using real-world data. In the strong condition, the share of reusable cups increased from 40.9% to 71.1% during the intervention and remained at 63.8% 3 weeks later. However, in the weak condition, there was a backfire effect: The use of reusable cups decreased from 21.3% to 5.5% and was only 1.9% after the intervention. Therefore, the backfire effects were evident but only in the case of a weak descriptive minority norm. It may be possible that a negative spiral occurred when individuals recognized a negative trend after the first week. However, if a positive trend occurred, it was further reinforced until the desired behavior exceeded the threshold for the majority behavior (>50%).

Kormos et al. (2015) arrived at a similar conclusion a few years earlier. In their field experiment, the participants were randomly assigned to a strong (26% changed their behavior) minority DSNA condition, a weak (4% changed their behavior) minority DSNA condition, or a control group. No differences were found among the conditions at the end of the intervention. However, they found a linear trend within the intervention weeks: The amount of sustainable transportation increased from the control group to the weak minority DSNA and then to the stronger minority DSNA. Kormos et al. (2015) discussed these results as partly surprising and in contrast with those observing backfire effects for minority behavior. Nevertheless, their study differed from previous ones in that it not only reported the proportion of individuals who engaged in the desired behavior at the time but also indicated a change (“Since 1993, 26% of commuters at [our university] have switched to more sustainable modes of transport to campus”). Thus, they came to a conclusion similar to that of Berger (2021), suggesting that the perception of change could lead to an adjustment of behavior. This idea about preconformity is the basis of studies that used dynamic DSNA to prevent the backfire effects of static DSNA.

#### Q4: How can dynamic descriptive social norm appeals prevent negative backfire effects by highlighting a trend in the behavior?

Sparkman et al. studied perceived social change and dynamic DSNAs extensively. Sparkman and Walton (2017) found a higher interest in reducing meat consumption using a dynamic minority DSNA (“in the last 5 years, 30% have started to change their behavior”) than a static DSNA (“30% make an effort”). When they added a control condition without social norm appeal, they did not find differences between the social norm appeal conditions and the control group. However, descriptively, the control group fell between the social norm appeal conditions, indicating a backfire effect of static minority DSNAs. When they added another dynamic condition and the trend was expected to either continue in the near future or not, the interest was higher in the condition suggesting future growth than in the condition without future growth.

In subsequent years, Sparkman et al. (2020b) conducted additional field experiments using dynamic DSNAs without specifying a baseline frequency (e.g., “customers are starting to eat less meat”) and found modest positive effects of the dynamic DSNAs. Moreover, Sparkman et al. (2021) found that a dynamic DSNA was able to shift the intention to reduce meat consumption for 5 months (Sparkman et al., 2021).

Using a similar approach, Mortensen et al. (2019) examined trending norms. They investigated whether the undesirable backfire effects of static minority DSNAs could be counteracted by highlighting a trend (48%; “this has increased from 37%”). A dynamic minority DSNA (48%; “this has increased from 37%”) caused significantly less water use than a static minority DSNA. Moreover, a marginal backfire effect of static minority DSNA was found and the water use was lower in the control condition than in the static condition. Furthermore, Loschelder et al. (2019) studied whether dynamic minority DSNAs could prevent the backfire effects of static minority DSNAs (25%). They described the trend without numbers and only stated that “more and more” people were changing their behavior. This dynamic DSNA had the strongest effect and performed significantly better than the static DSNA or control condition.

However, there are other studies that did not find positive effects of dynamic DSNAs. For example, Aldoh et al. (2021) replicated the study by Sparkman and Walton (2017) conceptually and compared static vs. dynamic DSNA (without future growth) with a control condition. However, they did not find any significant effects on the dependent variables. Descriptively, the interest in reducing meat consumption was lowest in the control group; thus, no backfire effects were found. Moreover, Schorn and Wirth (2023, 2024) and Chung and Lapinski (2023) did only find an indirect effect of the dynamic vs. static DSNA on behavioral intention when using perceived future descriptive norms as a mediator. Boenke et al. (2022) showed that a dynamic vs. static DSNA only led to higher intentions to reduce meat consumption when communicated by a researcher but not by a company representative or a vegan activist. They concluded that dynamic DSNA could backfire. However, since they did not include a control group in their (non-factorial) design, it was difficult to determine whether the dynamic DSNAs backfired or were just less effective when communicated by partisan people, because both static and dynamic DSNAs led to higher intentions when communicated by a researcher. In terms of red meat consumption and flu vaccinations, Lee and Liu (2023) did not find differences between static (30%/35%) and dynamic DSNAs without indicating future growth (cf. Sparkman and Walton, 2017). Nevertheless, they complemented their DSNA with a direct appeal (e.g., “get your flu shot”), which had already led to considerably smaller differences between dynamic and static DSNAs in Loschelder et al.'s (2019) study. However, Buvár et al. (2023), Carfora et al. (2022), Gossen et al. (2023), and Sparkman et al. (2020a,b) did also not find a positive effect of a dynamic DSNA. In particular, when comparing dynamic DSNAs against control groups other than static minority DSNAs, dynamic DSNAs may to be less effective (e.g., DellaValle and Zubaryeva, 2019; He et al., 2019).

Taken together, dynamic DSNAs seem to be a promising approach to promote minority behavior, especially when indicating ongoing future growth. However, the positive effects could have been overestimated, especially at the beginning, and the effects are now being modified with additional research. Specifically, when comparing dynamic DSNAs with a control group without social norm appeals (compared with static minority DSNAs), dynamic DSNAs seem to be less effective (Buvár et al., 2023; DellaValle and Zubaryeva, 2019; He et al., 2019; Carfora et al., 2022). Nevertheless, field experiments (Kormos et al., 2015; Loschelder et al., 2019) have suggested that dynamic DSNAs could be particularly effective over a longer period because they could then develop their full effect. Nevertheless, dynamic DSNAs appear to be preferable to static minority DSNAs, as backfire effects are unlikely, even if they may not effectively promote sustainable minority behavior.

### Effects of injunctive social norm appeals

Aside from using dynamic DSNAs, another strategy to prevent backfire effects is to highlight and activate injunctive norms (Schultz et al., 2007). The first finding was that all studies on minority behavior applied ISNAs that described the approval within a reference group (approving ISNAs) and not ISNAs that described that the behavior should be performed or that a reference group expects the behavior (prescriptive ISNAs; Schorn et al., 2023). Nevertheless, some studies on minority behavior were excluded from the process because they used direct behavioral appeals without stating an injunctive norm (e.g., Loschelder et al., 2019).

#### Q5: How do the effects of injunctive social norm appeals depend on the strength of the social norm appeal?

In an early study on the topic, Schultz et al. (2008) described that “some” vs. “many” people supported sustainable behavior. However, they did not find significant differences between the two groups. Similarly, Smith et al. (2012) did not find a main effect on behavioral intention based on their manipulation of approval regarding energy conservation measures in two studies (23% vs. 85%). de Groot and Schuitema (2012) found that the acceptability of a measure was higher when the behavior had been approved by a majority (80%−89%) than by a minority (10%−20%), but they did not measure persuasive outcomes. Moreover, there is a very recent study by Liu and Lapinski (2024) that compared minority (“only a few”) and majority ISNA (“the majority”). They did not find direct effects of ISNA on the behavioral intention, but an indirect effect mediated by perceived injunctive norms. However, they not only manipulated ISNAs, but also DSNAs (although the interaction was not taken into account in the analyses) and the effects of ISNAs can therefore not be assessed independently of the effects of DSNAs.

Lalot et al. (2019) conducted studies in the context of conversion theory (Moscovici, 1980). They described that a numerical minority (4%−18%) or majority (61%–82%) declare support and intent to make individual efforts, which is why they appeared to use a mix between ISNAs and DSNAs. Such a majority social norm appeal increased behavioral intentions. However, a minority social norm appeal and the control condition (no social norm appeal) only had a positive effect on people who engaged in green behavior in the past but not on those who reported less green behavior in the past. In another study, Lalot et al. (2018) found that a minority social norm appeal could be even more effective than a majority appeal when making participants feel good about their own environmental behavior. When making participants feel less good about their own environmental behavior, the majority social norm appeal increased willingness to participate in a pro-environmental event. Therefore, the participants who were led to believe that their behavior was insufficient were more willing to compensate for that when they believed that a majority (vs. minority) supported environmental values. Conversely, the participants who were led to believe that their behavior was sufficient maintained their efforts only when they believed that a minority supported those values, while self-licensing occurred when the majority supported those values. Therefore, Lalot et al. concluded that (injunctive) social norm appeals had different effects depending on individuals' environmental dispositions. One explanation for the opposing results to those of research on DSNAs, could be that their appeals may rather constitute ISNAs than DSNAs (because support and intentions were described): Different DSNAs could have stronger effects on individuals with weak environmental dispositions, while different ISNAs could have stronger effects on individuals with strong environmental dispositions (cf. Lalot et al., 2018).

Taken together, research suggests that ISNAs are less prone to backfire effects than DSNAs when promoting sustainable behavior and there were little differences between majority and minority ISNAs. Nevertheless, the studies comparing minority and majority ISNAs did typically not include control groups without an ISNAs which means that it is hard to tell if the minority and majority ISNAs may be equally effective or ineffective. Moreover, in most studies in which ISNAs were manipulated, DSNAs were manipulated as well. For example, Schultz et al. (2008) and Smith et al. (2012) who did not find main effects of ISNAs, found interaction effects between injunctive and DSNAs.

### Effects of the alignment of social norm appeals

Only a few studies have been conducted on the alignment of social norm appeals (see Table A1). The studies differ in that in some cases no full design was used (e.g., no minority ISNA in Schorn and Wirth, 2023, 2024) or not all combinations were statistically analyzed (e.g., Schultz et al., 2008; Liu and Lapinski, 2024). In addition, numbers were used for DSNAs in some studies, while the proportion in ISNA was vaguely described (e.g., Schultz et al., 2008; Liu and Lapinski, 2024). Other studies used numbers for ISNAs and DSNAs (e.g., Smith et al., 2012).

#### Q6: How does the alignment of descriptive and injunctive social norm appels affect their impact?

Schultz et al. (2008) combined a majority (“many”) vs. a minority (“some”) ISNA with a DSNA and determined whether a majority (75%) vs. a minority (25%) reused their towels. They showed a significant difference between the aligned majority social norm appeals and all other conditions, with the aligned majority social norm appeals being the most effective. Unfortunately, they reported only the results for this contrast. Similarly, Liu and Lapinski (2024) combined minority (“a few”) and majority (“the majority”) ISNAs with minority (20%) and majority (80%) DSNAs. Although the effects of ISNAs and DSNAs can therefore not considered independently, no interaction effects on persuasive outcomes were reported. The results of the manipulation checks showed no interaction effects, but weak spillover effects between the social norms in addition to the expected effects: ISNAs had a weak effect on perceived descriptive norms and DSNAs had a weak effect on perceived injunctive norms.

Moreover, as previously mentioned, Smith et al. (2012) did not find main effects for majority vs. minority descriptive and ISNAs, but they found an interaction: When a majority DSNA (82%) was combined with a majority ISNA (85%), the intentions to conserve energy were higher than when a majority DSNA was complemented with a minority ISNA (23%) or when a majority ISNA was paired with a minority DSNA. However, when using a minority ISNA, no significant differences were found between the descriptive majority and minority social norm appeals. In sum, the participants in the aligned majority social norm condition reported stronger intentions to engage in energy conservation than did the participants in either the unaligned conditions or the aligned minority social norm appeal condition.

Nevertheless, recent studies by Schorn and Wirth (2023, 2024) did not find indications of social norm conflict when combining a majority ISNA (80%) with a minority DSNA (10%). However, unlike the other studies, they did not vary the strength of the ISNA but only compared the presence or absence of a majority ISNA in combination with a static, dynamic, or no DSNA. However, similar to Liu and Lapinski (2024), they found effects on perceived social norms: There were not only the expected main effects of DSNA and ISNA but also spillover effects and the majority ISNA proved to be particularly beneficial, as it had a desirable influence on both perceived injunctive and descriptive norms, as long as the prevalence of the behavior was not explicitly mentioned (no minority DSNA including a baseline). Minority DSNAs were also able to influence perceived injunctive norms, but this was a disadvantage in the case of minority behavior. Even though no direct effects on behavioral intention were found, the results of a mediation analysis suggest that DSNAs and ISNAs can indirectly influence behavioral intention via perceived social norms (Schorn and Wirth, 2024). Moreover, there were interaction effects between DSNA and ISNA that suggest that majority ISNAs can prevent the negative effects of minority DSNAs on perceived norms, but their positive effect on perceived injunctive norms and persuasive outcomes is not diminished by minority DSNAs. Schorn and Wirth (2024) conclude that possible negative effects of conflicting social norm appeals cannot be explained by the effects of DSNA and ISNA on perceived social norms but must have other origins. Nevertheless, they did not include minority ISNAs in their study, and due to incomplete research design, only limited statements can be made on how these effects on perceived social norms explain (absent) effects on behavioral intentions. Smith et al. (2012) demonstrated that the combination of majority DSNA and majority ISNA was the most effective and derive the negative effects of conflicting norm appeals from the contrast to this condition—a combination that was not investigated by Schorn and Wirth (2023, 2024).

Taken together, it is still not clear if the combination of (majority) ISNAs and (minority) DSNAs is problematic in the context of sustainable behavior. Smith et al. (2012) and Schultz et al. (2008) found interactions between DSNAs and ISNAs when using students or hotel guests as rather narrow reference group. Schorn and Wirth (2023, 2024) did not find interaction effects but they used the German population as rather broad reference group and did not include minority ISNAs. When extending the view to areas other than sustainable behavior, there have been studies on organ donations that show even positive effects of conflicting social norm appeals (Habib et al., 2021). Therefore, more research on conflicting social norms is needed and researchers should also include perceived social norms to be able to provide insight into the mechanisms of operation of conflicting social norms and norm appeals, which may explain positive and negative effects.

## Discussion

In this review, 36 articles, including 54 studies, applying social norm appeals to promote sustainable minority behavior were reviewed and discussed. Overall, there has been an increased number of studies on social norm appeals to promote sustainable behavior performed by a numerical minority of people. Most studies have indicated that minority DSNAs are not effective in promoting sustainable behavior (e.g., Richter et al., 2018; Shealy et al., 2018; Berger, 2021). Moreover, some indicated that the use of static minority DSNAs was unpredictable and could backfire (Richter et al., 2018; Mortensen et al., 2019; Berger, 2021). However, environmental dispositions of the population could play a significant role in the outcome (Demarque et al., 2015; Richter et al., 2018; de Groot et al., 2021; Aruta, 2022). It appears that DSNAs have a stronger effect on communities with lower pro-environmental attitudes or in populations with a lower baseline level of sustainable behaviors. People with higher pro-environmental attitudes seemed to be less affected. Results may be reversed for ISNA (cf. Lalot et al., 2018) but there is a need for further research because these results are partly implicit, explorative, or not robust which is why studies are necessary to clarify if cultural or environmental dispositions have a relevant effect on the impact of (minority) social norm appeals.

When a trend in the minority behavior was highlighted, most studies revealed positive effects (e.g., Sparkman and Walton, 2017; Loschelder et al., 2019; Mortensen et al., 2019; de Groot, 2022). However, it appears that dynamic DSNAs were more likely to catch backfire effects from static minority DSNAs, as the overall results were weaker when dynamic DSNAs were compared against the control groups without social norm appeals (e.g., Buvár et al., 2023; DellaValle and Zubaryeva, 2019; Carfora et al., 2022; Gossen et al., 2023). Nevertheless, dynamic DSNAs may be particularly effective over a longer period because change can then be experienced, and they can develop their full effect (e.g., Kormos et al., 2015; Loschelder et al., 2019; Berger, 2021, study 1). In line with that, several studies have suggested that the effect of dynamic DSNAs is mediated by preconformity or perceived future descriptive norms (e.g., Loschelder et al., 2019; Chung and Lapinski, 2023; Schorn and Wirth, 2023). Future studies should therefore look more closely at how dynamic DSNAs work over a longer period of time and what effect the adjustment of dynamic DSNAs has within this period. In this context, for example, effects in social media could also be considered and it could be examined whether algorithms affect social norms and reinforce the effects of and social norm appeals (Lutkenhaus et al., 2023; Schorn and Wirth, 2024).

In addition, there could be differences depending on the wording or presentation of the trend (cf. Sparkman and Walton, 2017; de Groot et al., 2021; de Groot, 2022). More research is necessary to determine if these differences are crucial for the persuasive effects and for example, it could be relevant if the trend is expected to continue in the future or not (cf. Sparkman and Walton, 2017). Additionally, dynamic DSNAs may be more effective if they include numbers, and the trend is not only described vaguely (“more and more”). Numeric DSNAs could be more credible than vague DSNAs although there may be no differences regarding persuasive effects (Schorn, 2023).

Furthermore, it has not yet been investigated in the context of sustainable behaviors whether dynamic DSNAs have a similar effect or may be even more effective than majority DSNAs. Chung and Lapinski (2023) included dynamic minority DSNAs and static majority DSNAs but only reported the effects mediated by perceived descriptive future norms. Nevertheless, a dynamic DSNA (an increase from 9% to 30%) led to a higher perceived future descriptive norm than a static majority DSNA (65%), which had a positive effect on behavioral intentions. However, they only found this effect for unplugging electronic devices but not for bringing one's own bags for grocery shopping to reduce plastic waste. Moreover, a very recent study by Zumthurm and Stämpfli (2024) used a dynamic DSNA which described the shift from minority to majority behavior (“In Switzerland, more and more people are reducing their meat consumption. Whereas 10 years ago, it was 40 % of the population that occasionally refrained from meat, today it is 60 %, which have adjusted their eating habits and occasionally refrain from meat”). They did not find significant differences to the control group without an appeal—although 60% is even majority behavior.

In addition to dynamic DSNAs, (majority) ISNAs can be used to prevent the backfire effects of (static) minority DSNAs because they seem to be less prone to backfire effects (Schultz et al., 2008). There were little differences between majority and minority ISNAs, but the studies typically did not include control groups without an ISNAs which means that it is hard to tell if the minority and majority ISNAs are equally effective or ineffective (e.g., de Groot and Schuitema, 2012). However, Schorn and Wirth (2023, 2024) conclude that majority ISNAs can have a positive effect in the context of minority behavior, but they only compared a majority ISNA to the control group. Nevertheless, minority ISNAs may be effective because individuals could spend more attention on measures supported by a few people, and this could lead to a stronger internalization of reasons for engaging in the behavior (Lalot et al., 2019). Unlike DSNAs that typically work heuristically through the peripheral route of information, ISNAs need more elaboration to make the “right” choice (Göckeritz et al., 2009; Melnyk et al., 2019). This conscious decision can be more stable and can have an impact on different future situations. Therefore, when individuals think about good motives to engage in behavior supported by a minority of people, a resulting agreement with the minority position could increase people's motivation to adopt the behavior (Lalot et al., 2019). Nevertheless, open questions remain specifically about the effectiveness of ISNAs stating majority approval in the context of minority behavior because most of the studies combined ISNAs with DSNAs.

When looking at studies on conflicting social norms, the results are ambiguous. In early studies, it was problematic when a majority ISNA did not align with a majority DSNAs (e.g., Smith et al., 2012). When looking at the greater picture, these results suggest that majority ISNAs may be fragile because even if the descriptive minority norm is not made salient in the appeal, people still have an idea about whether the behavior is performed in general, as they quite accurately infer social norms through their observation of others, personal and media communication, and self-knowledge (Cialdini et al., 1991; Miller and Prentice, 1996; Witzling et al., 2019; Griesoph et al., 2021). Survey studies have shown that such perceived norms strongly influence behavior (e.g., Borg et al., 2020; Jacobson et al., 2020). Even if the injunctive norm was perceived as strong, which could be reinforced through the majority ISNA, it was problematic when it did not align with the perceived descriptive norm because the effect of an ISNA could be moderated through perceived descriptive norms (Thøgersen, 2008; Witzling et al., 2019; Jacobson et al., 2020). In this case, people could experience an inner conflict or cognitive dissonance, which could suppress the desired behavior (cf. Thøgersen, 2008; Jacobson et al., 2020). Especially when a behavior involves effort, people may question why they should act when no one else does. As sustainable behavior often represents a social dilemma, individuals may have no direct benefit but have costs and effort instead (Thøgersen, 2008).

Conversely, Schorn and Wirth; Schorn and Wirth (2023; 2024, study 1) did not find undesirable effects caused by a social norm conflict or nullification of the main effects when combining a majority ISNA with a static or dynamic DSNA. However, they discussed whether this could be caused by the online environment because a majority ISNA showed the participants the “right” thing to do, and as there was no cost to providing that answer, the participants might do so. On the one hand, it can be argued that social desirability is of minor importance in an online setting, because the behavior is anonymous and not publicly visible. On the other hand, the effect of social norm appeals may have been weakened precisely by the fact that the actions were not publicly visible, but the behavior was carried out in private. Social norm appeals may have stronger impacts in public contexts because such contexts may increase the influence of social proof and social pressure and therefore the sensitivity to norm manipulations (Habib et al., 2021).

Nevertheless, in health communication, Habib et al. (2021) even came to the conclusion that a minority DSNA in combination with a majority ISNA could result in greater organ donor registrations than either of them separately. This could be due to the discrepancy between what people think they should do and what they actually do becoming the most salient. However, organ donation could have direct consequences for one individual, whereas sustainable behavior would only be effective if it was implemented by a sufficiently large number of people. At the same time, individuals could run along in this crowd without doing anything themselves when a sufficient majority engages in sustainable behavior (Thøgersen, 2008; Lalot et al., 2019). Moreover, there may have been a shift in times with regard to sustainable behavior because media reporting on the climate crisis has significantly changed since the early studies on social norm conflict and the topic is now more relevant (cf. Smith and Louis, 2008; McDonald et al., 2014). Following this line of argumentation, the injunctive majority approving the behavior may now be stronger manifested in society and an attitude–behavior gap appear more legitimate because structural measures are often demanded instead of changing one's own behavior. When reminding individuals that a behavior is approved by a majority but yet only performed by a minority of people, this could be a strong motivator because social rewards may be particularly attractive. In this case, the adoption of the behavior would be rather driven by social rewards than by the fear of social sanctions. Furthermore, studies addressing sustainable behavior often used topics that are not relevant to the single individual, and in this case, social norm appeals could operate heuristically and without deep elaboration (Smith and Louis, 2008). If the topic was personally relevant (cf. Habib et al., 2021) or explained in detail (Schorn and Wirth, 2023), individual group members might feel an obligation or a stronger motivation to engage in the course of action when no one else does.

To provide further insight into the effect of conflicting social norm appeals, future research could examine if there are differences depending on the formulation of conflicting social norm appeals. Most studies using DSNAs have focused on statistics or numeric information, while some of the studies used vague wording for ISNAs. Nevertheless, there is an increasing number of studies that use vague formulations of a trend as dynamic DSNA (Bergquist and Nilsson, 2019; Bergquist et al., 2020; Loschelder et al., 2019, study 1; Schultz et al., 2008). Within social norm conflicts, the injunctive majority could be emphasized in a numeric ISNA combined with vague wording for the minority DSNA to mitigate the perception of incongruent injunctive and descriptive norms. Moreover, majority ISNAs could be combined with dynamic DSNAs. Schorn and Wirth (2023, 2024) did not find positive effects of combining majority ISNAs with dynamic DSNAs, but studies on this combination are limited to date. Furthermore, instead of highlighting the increasing minority performing the behavior (e.g., increase to 30%), social norm appeals could highlight the decreasing majority (e.g., decrease to 70%) not engaging in the target behavior (de Groot, 2022). Finally, the combination of dynamic majority ISNAs and static minority DSNAs could be investigated (e.g., “an increasing majority supports the behavior, although only a few perform the behavior yet”).

Overall, there is still a need for further research investigating social norm appeals to promote sustainable minority behavior approved by most people. This research should particularly focus on interventions in real-world settings and investigate how they can influence perceived social norms over extended periods and, consequently, effectuate lasting behavioral changes. Such studies may consider the combination of majority ISNAs with dynamic DSNAs or vaguely formulated social norm appeals, as these approaches appear to have potential. In addition, it should be further investigated under which conditions social norm appeals are effective in the long term and what role individual characteristics, such as environmental concerns or personal norms, play in this.

## Conclusion

This literature review discussed studies that use social norm appeals in the context of sustainable minority behavior. It is striking that most studies refer to descriptive norms and only a few considered injunctive norms, although the combination is very relevant, especially in the area of sustainable behavior. Overall, it is not yet clear how effective social norm appeals are in promoting minority behavior but it is worthwhile to investigate social norm appeals in this context: They are typically easy to implement without incurring high costs and according to conversion theory, minority influence is the “true” influence, while majority influence is superficial (Moscovici, 1980). Therefore, social norm appeals could result in stronger and more stable changes in attitudes and behavior if they do not backfire (Lalot et al., 2019). Under specific circumstances, minority social norm appeals may even increase the urgency to act when individuals realize how critical the issue is (Habib et al., 2021). Nevertheless, the activation and adjustment of (perceived) injunctive majority norms appears to be especially effective because they are often underestimated and majority ISNAs can not only adjust such misperceptions but also have positive spillover effects on perceived descriptive norms (Schorn and Wirth, 2024). Therefore, for practitioners, emphasizing ongoing social change toward the desired behavior and highlighting majority approval seem to be simple strategies with great potential to induce compliance and encourage sustainable minority behavior without running the risk of undesirable backfire effects.

## Appendix

**Table A1:** List of studies selected for the literature review.

**References**	**Study**	**Method**	**Topic**	**Country**	**DSNA**	**ISNA**	**Control group**	**Exemplar minority appeal**	**Stimulus**
Aldoh et al. (2021)	–	Experiment	Sustainable diet (meat consumption)	United Kingdom	30% vs. 30% (increasing) vs. control group	–	No stimulus	Recent research has shown that, in the last 5 years, 30% of people in the UK have now started to make an effort to limit their meat consumption.	Statement
Aruta (2022)	Study 2	Experiment	Waste prevention (plastic)	Philippines (students)	20% vs. 80%	–	–	Among these institutions, our university only has 20% of its students minimizing their daily plastic use.	Article
Berger (2021)	–	Field experiment	Waste prevention (reusable mugs)	Switzerland (students)	40.9% and 21.3%	–	Behavioral appeal No intervention	1st week of October. 22% are using mugs. Join them!	Sign
Boenke et al. (2022)	–	Experiment	Sustainable diet (meat consumption)	Germany, Switzerland, and Austria (convenience)	15% vs. more and more	–	–	15% of Germans try to reduce their meat consumption. This means that 15 out of 100 people eat less meat than they would normally do.	Statement
Buvár et al. (2023)	–	Experiment	Waste prevention (plastic)	Finland (convenience)	More and more vs. control group	–	Behavioral appeal	More and more people care about the PET pollution every day.	Instagram post
Carfora et al. (2022)	–	Experiment	Sustainable diet (meat consumption)	Italy (convenience)	More and more vs. control group	–	No social norm appeal	To avoid deforestation, more and more people are reducing their consumption of meat.	Chatbot
Chung and Lapinski (2023)	–	Experiment	Waste prevention Conservation (energy)	USA (students)	30% vs. 30% (increasing) vs. majority	–	–	More and more students (9% in 2017 → 17% in 2018 → 30% in 2019) are unplugging their electronics to save money, save energy, and to protect pure Michigan.	Poster
de Groot (2022)	–	Experiment	Sustainable diet (meat consumption)	Germany The Netherlands (students)	30% vs. 30% (increasing)	–	No stimulus	Recent research has shown that 30% of people living in the Netherland make an effort to limit their meat consumption. This means that 3 out of 10 people living in the Netherlands eat less meat than they otherwise would. This has increased from 20% or 2 out of 10 people five years ago.	Statement
de Groot and Schuitema (2012)	–	Experiment	Waste prevention Transportation	UK (convenience)	–	10%−20% vs. 80%−90%	–	A pilot survey indicated that around 10%/90% of a representative sample of the UK population found that this was an acceptable measure for reducing car use.	Article + statement
de Groot et al. (2021)	Study 2	Experiment	Sustainable diet (meat consumption)	The Netherlands (convenience)	20% vs. 80%	–	–	Around 80%/20% of the Dutch population is either trying, or considering to make an effort to limit the amount of meat they consumer.	Article
DellaValle and Zubaryeva (2019)	–	Experiment	Transportation	Italy (convenience)	Increase by 178% vs. control group	–	No social norm appeal No stimulus	South Tyroleans increased in new Electric Vehicle registrations by 178 % in the period 2013–2017.	Ad
Demarque et al. (2015)	Study 1	Experiment	Sustainable consumption	France (students)	9% vs. 70% vs. control group	–	No social norm appeal	For your information, 9% of previous participants purchased one ecological product.	Online shop
Demarque et al. (2015)	Study 2	Experiment	Sustainable consumption	France (students)	1% vs. 9% vs. 70% vs. 90% vs. control groups	–	No social norm appeal	For your information, 9% of previous participants purchased one ecological product.	Online shop
Gossen et al. (2023)	–	Experiment	Sustainable consumption	Germany (quasi representative)	More and more vs. control group	–	No social norm appeal	In collaboration with scientists, we have found that more and more people are extending the life of their mobile phone by getting it repaired.	Online shop
Griesoph et al. (2021)	–	Field experiment	Sustainable diet (meat consumption)	Germany (students)	Positive (44% vegan) vs. negative (56% meat eaters) vs. control group	–	No social norm appeal	On average, 44% of our canteen customers chose a vegan or vegetarian main dish during the last winter term.	Statement
He et al. (2019)	Study 1–3	Experiment	Conservation (energy)	China (students + convenience)	More and more vs. control group	–	Appeal (every student should)	More and more university students set the air conditioning temperature above 26°C in the summer and below 20°C in the winter.	Poster
Kácha and van der Linden (2021)	Study 1–2	Experiment	Pro-environmental behavior	USA (quasi representative)	17% vs. 83%	–	–	17% of other participants have completed the task.	Statement
Kormos et al. (2015)	–	Field experiment	Transportation	Canada (students)	4% vs. 25% vs. control group (no social norm appeal)	–	No social norm appeal	Since 1993, 26% of commuters at [our university] have switched to more sustainable modes of transport to campus vs. only 4% of commuters at [our university].	Statement
Lalot et al. (2018)	Study 1	Experiment	Sustainable consumption	USA (MTurk)	–	18% vs. 82%	–	18% [82%] of the individuals declared supporting the content of the text without hesitation, and committed to make more individual efforts in order to reduce their own consumption. Thus, only a minority [a large majority] of the inhabitants supports unconditionally proenvironmental values.	Article
Lalot et al. (2018)	Study 2	Experiment	Sustainable consumption	Switzerland (students)	–	12% vs. 88%	–	12% [88%] of the individuals declared supporting the content of the text without hesitation, and committed to make more individual efforts in order to reduce their own consumption. Thus, only a minority [a large majority] of the inhabitants supports unconditionally proenvironmental values.	Article
Lalot et al. (2019)	Study 1	Experiment	Waste prevention (recycling)	USA (MTurk)	–	4% to 18% vs. 61% to 82%	–	[Most/Few] Americans are concerned for the environment, view protecting the environment as a top priority, say they try to live in ways that protect the environment; and [a majority/a minority] of Americans do recycle.	Article
Lalot et al. (2019)	Study 2	Experiment	Sustainable consumption	Switzerland (students)	–	12% vs. 88%	–	“12% (88%) of the sampled people declared supporting the content of the text without hesitation, and committed themselves to make more individual efforts in order to reduce their own consumption. Thus, only a minority (a large majority) of the inhabitants.	Article
Lapinski et al. (2017)	–	Experiment	Conservation (water)	USA (students)	3% vs. 90%	–	–	Only 3% of people in the university community took steps to conserve water in the year prior to the study.	Statement
Liu and Lapinski (2024)	–	Experiment	Food waste (students)	China USA (students)	20% vs. 80%	Few vs. majority	–	Only a few of students at XX University, about 20%, have taken actions to help reduce food waste.	Article
Lee and Liu (2023)	–	Experiment	Sustainable diet	USA (students)	35% vs. 35% increased vs. control	–	No stimulus	Recent research has shown that, in the last 5 years, 35% of Americans have now started to make an effort to eat less meat.	Statement
Loschelder et al. (2019)	Study 1	Field experiment	Reusable mugs	Germany (students)	More and more vs. control group	–	No social norm appeal	Our guests are changing their behavior: More and more are switching from the to-go-cup to a sustainable alternative	Sign
Loschelder et al. (2019)	Study 2	Experiment	Reusable mugs	Germany (students)	25% vs. more and more	–	No social norm appeal Behavioral appeal	Our guests are changing their behavior: More and more are switching from the to-go-cup to a sustainable alternative.	Sign
Mortensen et al. (2019)	Study 1	Experiment	Water conservation	USA (students)	48% vs. increase from 37% to 48%	–	Architectural trends	Research from (previous year) has found that 48% of (University name) students engage in one or more of the following water conservation behaviors. This has increased from 37% in (2 years previous).	Statement
Mortensen et al. (2019)	Study 2	Experiment	Donations	USA (MTurk)	48% vs. increase from 17% to 48%	–	No social norm appeal	In July [previous year], 48% of the MTurk workers who took our surveys donated funds to the SEAA. This increased from 17% in July (2years previous).	Statement
Player et al. (2018)	–	Field experiment	Idling engines	UK (convenience)	25% vs. negative (some don't)	–	–	When barriers are down 25% of motorists turn off their engines!	Sign
Reynolds-Tylus et al. (2019)	–	Experiment	Conservation	USA (MTurk)	27% vs. 73% 32% vs. 68%	–	–	27% of Americans take small steps to conserve their water use.	Statement
Richter et al. (2018)	Study 1	Field experiment	Sustainable diet	Germany Norway (convenience)	1% vs. 4% vs. 28% vs. 52% vs. 69% vs. 82% vs. 91%	–	No social norm appeal No intervention	11% of all customers buying seafood in our shop yesterday chose MSC/ASC.	Sign
Rinscheid et al. (2021)	–	Choice experiment	Fossil fuel-powered cars	USA (quasi representative)	Few vs. more and more	–	–	More and more people living in [your state] are buying non-fossil fuel cars and have already started to change their transportation habits, e.g. by using public transportation.	Article + statement
Schorn and Wirth (2023)	Study 1	Experiment	Voluntary carbon offsets	Germany (quasi representative)	Minority vs. increase of 50%	–	No social norm appeal No stimulus	Unfortunately, so far only a minority compensates their CO2 emissions. But there is good news: More and more people are offsetting their emissions.	Video
Schorn and Wirth (2023)	Study 2	Experiment	Voluntary carbon offsets	Germany (quasi representative)	10% vs. 10% increasing vs. none	80% approval vs. none	No social norm appeal No stimulus	Already one in ten people voluntarily offset their flight in 2021. That is five times more people than in the previous year. So there is a positive trend and the proportion is expected to quadruple in the next few years.	Video
Schorn and Wirth (2023)	Study 1	Experiment	Voluntary carbon offsets	Germany (quasi representative)	10% vs. 10% increasing vs. none	80% approval vs. none	No social norm appeal No stimulus	When looking at the German population, already one in ten people voluntarily offset their flight in 2020. This number is five times higher than in the previous year. This is a positive trend, and the proportion is expected to quadruple in the coming years.	Video
Schorn and Wirth (2023)	Study 2	Experiment	Voluntary carbon offsets	Germany (quasi representative)	10% vs. more and more vs. none	80% approval vs. none	No social norm appeal No stimulus	A strong trend is emerging: More and more people are choosing to offset their air travel.	Video
Schultz et al. (2008)	Study 1	Field experiment	Reusing towels	USA (convenience)	25% vs. 75%	Some vs. many	No social norm appeal	Nearly 25% of guests choose to reuse their towels each day. Some of our guests have expressed to us their approval of conserving energy. Because some guests value conservation, this hotel has initiated a conservation program.	Sign
Shealy et al. (2018)	–	Experiment	Sustainable building	USA (convenience)	6-20% vs. 80-95%	–	–	Less than 20 % of Envision projects are able to meet the Restorative level of achievement for this credit.	Statement
Smith et al. (2012)	Study 1	Experiment	Energy conservation	UK (students)	82% vs. 22%	85% vs. 23%	–	Participants were told that 22% of students engaged in energy conservation.	Article
Smith et al. (2012)	Study 2	Experiment	Energy conservation	China UK (students)	82% vs. 22%	85% vs. 23%	–	Participants were told that 22% of students engaged in energy conservation.	Article
Sparkman et al. (2021)	Study 1–2	Experiment	Sustainable diet	USA (MTurk, quasi representative)	More and more vs. control group	–	No social norm appeal	In the US, over six million people have reduced their meat intake, and that number is rising.	Article
Sparkman and Walton (2017)	Study 1–2	Experiment	Sustainable diet	USA (MTurk)	30% vs. 30% (increasing)	–	No social norm appeal	Recent research has shown that, in the last 5 years, 30% of Americans have now started to make an effort to limit their meat consumption.	Statement + visualization
Sparkman and Walton (2017)	Study 3	Experiment	Sustainable diet	USA (MTurk)	30% vs. 30% (increasing; future growth) vs. 30% (increasing; no future growth)	–	No social norm appeal	Recent research has shown that, in the last 5 years, 30% of Americans have now started to make an effort to limit their meat consumption. […] This trend is expected to continue in the near future.	Statement + visualization
Sparkman and Walton (2017)	Study 4	Field experiment	Sustainable diet	USA (students)	30% vs. 30% (increasing) vs. description of an off-topic trend (control group)	–	No social norm appeal	Specifically, recent research has shown that 30% of Americans make an effort to limit their meat consumption. That means that 3 in 10 people eat less meat than they otherwise would.	Statement
Sparkman et al. (2020b)	Study 1	Field experiment	Sustainable diet	USA (convenience)	Dynamic vs. control group	–	No social norm appeal	Our Meatless Burgers Are on the Rise. We've noticed customers are starting to choose more meatless dishes.	Sign
Sparkman et al. (2020b)	Study 2–4	Field experiment	Sustainable diet	USA (convenience)	Dynamic vs. control group	–	No social norm appeal	We've noticed that customers are starting to eat less meat by choosing more meatless dishes.	Sign
